# Knockout of three aminopeptidase N genes does not affect susceptibility of *Helicoverpa armigera* larvae to *Bacillus thuringiensis* Cry1A and Cry2A toxins

**DOI:** 10.1111/1744-7917.12666

**Published:** 2019-03-14

**Authors:** Jing Wang, Ya‐Yun Zuo, Ling‐Li Li, Hui Wang, Shao‐Yan Liu, Yi‐Hua Yang, Yi‐Dong Wu

**Affiliations:** ^1^ College of Plant Protection Nanjing Agricultural University Nanjing China

**Keywords:** aminopeptidase N, *Bacillus thuringiensis*, CRISPR/Cas9, *Helicoverpa armigera*, receptor

## Abstract

*Bacillus thuringiensis* (Bt) insecticidal toxins have been globally utilized for control of agricultural insects through spraying or transgenic crops. Binding of Bt toxins to special receptors on midgut epithelial cells of target insects is a key step in the mode of action. Previous studies suggested aminopeptidase N1 (APN1) as a receptor or putative receptor in several lepidopteran insects including *Helicoverpa armigera* through evidence from RNA interefence‐based gene silencing approaches. In the current study we tested the role of APNs in the mode of action of Bt toxins using clustered regularly interspaced palindromic repeats (CRISPR)/CRISPR‐associated protein 9‐mediated gene knockout. Three APN genes (*HaAPN1*, *HaAPN2* and *HaAPN5*) were individually knocked out in a susceptible strain (SCD) of *H. armigera* to establish three homozygous knockout strains. Qualitative *in vitro* binding studies indicated binding of Cry1Ac or Cry2Ab to midgut brush border membrane vesicles was not obviously affected by APN knockout. Bioassay results showed that none of the three knockouts had significant changes in susceptibility to Cry1A or Cry2A toxins when compared with the SCD strain. This suggests that the three *HaAPN* genes we tested may not be critical in the mode of action of Cry1A or Cry2A toxins in *H. armigera*.

## Introduction

*Bacillus thuringiensis* (Bt) is a Gram‐positive bacterium that produces insecticidal crystal (Cry) proteins during sporulation (Schnepf *et al*., 1998). Due to high effectiveness against pests and safety to human beings and beneficial insects, Bt has been widely used for insect pest control in agriculture and public health, either as formulated biopesticides or in transgenic crops (Sanahuja *et al*., 2011). The mechanism of action of Bt Cry proteins against susceptible lepidopterans is complex and consists of multiple steps (Pigott & Ellar, 2007; Pardo‐Lopez *et al*., 2013). The inclusion body is solubilized in the larval midgut to release protoxins, which are then processed by gut proteases into active toxins. The toxins then bind to specific receptors at the surface of midgut epithelia cells, subsequently resulting in membrane insertion, pore formation and cell lysis (de Maagd *et al*., 2001). Although there is general agreement on the above main steps of mode of action, the precise mechanism of toxin‐receptor interactions and pore formation is still poorly understood.

A number of insect membrane proteins have been identified as receptors or putative receptors for Cry toxins. Such receptors include cadherins, adenosine triphosphate‐binding cassette (ABC) transporters, alkaline phosphatases (ALPs), and aminopeptidase Ns (APNs). A large amount of evidence from both forward and reverse genetics have confirmed that cadherins and ABC transporters are functional receptors for Cry1A or Cry2A toxins in some lepidopteran insects (Gahan *et al*., 2001, 2010; Tay *et al*., 2015; Wang *et al*., 2016, 2017). Based on correlation between RNA interference (RNAi)‐mediated knockdown of APN expression and reduced susceptibility to Cry toxins, several studies have suggested APNs are putative Bt receptors (Rajagopal *et al*., 2002; Sivakumar *et al*., 2007; Flores‐Escobar *et al*., 2013; Zhao *et al*., 2017).

The cotton bollworm, *Helicoverpa armigera*, is a serious polyphagous pest of global importance (Tay *et al*., 2013). Control of this pest used to rely on chemical insecticides, but it has been mainly dependent on transgenic cotton expressing Bt proteins since 1996 (Zhang *et al*., 2012). Due to intensive use of Bt cotton in China, resistance frequencies in field populations of *H. armigera* from northern China have significantly increased (Zhang *et al*., 2011, 2012; Jin *et al*., 2015, 2018). Evolution of insect resistance to Bt toxins has been a major threat to the long‐term effectiveness of Bt crops (Tabashnik & Carrière, 2017). Understanding the mode of action of Bt toxins and mechanism of Bt resistance in *H. armigera* is essential for developing resistance detection methods and designing adaptive resistance management strategies (Wu, 2014; Jin *et al*., 2018).

In *H. armigera*, mutations of the cadherin gene (*HaCad*) and the ABC transporter C2 gene (*HaABCC2*) are genetically linked to Cry1Ac resistance (Xu *et al*., 2005; Xiao *et al*., 2014), and mutations of the ABC transporter A2 gene (*HaABCA2*) are genetically linked with resistance to Cry2Ab (Tay *et al*., 2015). Using the CRISPR/Cas9 system (clustered regularly interspaced palindromic repeats/CRISPR‐associated protein 9), *HaCad* and *HaABCA2* have been validated as functional receptors for Cry1Ac and Cry2Ab, respectively (Wang *et al*., 2016, 2017). With respect to the functioning of *H. armigera* APNs (HaAPNs) as receptors for Bt Cry toxins, current evidence is still suggestive. Silencing of *HaAPN1* with RNAi caused reduced transcript levels and a corresponding decrease in the susceptibility of *H. armigera* larvae to Cry1Ac (Sivakumar *et al*., 2007). Knockdown of *HaAPN4* with RNAi significantly reduced the susceptibility of *H. armigera* larvae to both Cry2Aa and Cry1Ac (Zhao *et al*., 2017). Furthermore, a deletion mutation of *HaAPN1* was detected in the Cry1Ac‐resistant BtR strain of *H. armigera* (Zhang *et al*., 2009); however, genetic linkage between the mutated allele of *HaAPN1* and Cry1Ac resistance has not been established.

Our study aimed to investigate the involvement of *H. armigera* APNs in mediating Cry toxin susceptibility through CRISPR/Cas9‐based reverse genetics approach. We hypothesized that if an HaAPN functions as a receptor for a Cry toxin, knockout of this *HaAPN* will increase tolerance to the Cry toxin. Based on previous results (Sivakumar *et al*., 2007; Zhang *et al*., 2009), we selected *HaAPN1* as the priority target gene to knock out. *HaAPN2* and *HaAPN5* were also included in this study because *APN2* and *APN5* from other lepidopteran insects were reported to interact with Cry toxins (Pigott & Ellar, 2007). Thus, *HaAPN1*, *HaAPN2* and *HaAPN5* were individually knocked out in the susceptible SCD strain of *H. armigera* to establish three homozygous knockout strains. However, none of the three knockouts had significant changes in susceptibility to Cry1A or Cry2A toxins when compared with the background SCD strain.

## Materials and methods

### Insect strains

The background strain SCD, originally started from the Côte D'Ivoire (Ivory Coast), Africa has been maintained in the laboratory without exposure to insecticides or Bt toxins for over 30 years (Yang *et al*., 2009). The SCD strain is susceptible to both chemical insecticides and Bt toxins (Yang *et al*., 2009, 2013). Three APN genes (*HaAPN1*, *HaAPN2* and *HaAPN5*) were individually knocked out from the susceptible strain SCD. Three homozygous knockout strains were established and named as SCD‐APN1, SCD‐APN2 and SCD‐APN5.

Larvae of all the strains were reared on an artificial diet based on soybean flour and wheat germ (Yang *et al*., 2009) at 26 ± 1 °C, 60% ± 10% relative humidity (RH) and a photoperiod of 16 : 8 h (L : D). For adults, 10% sugar solution was supplied to replenish energy.

### Bt toxins and diet bioassays

Activated toxins, Cry1Aa, Cry1Ab and Cry1Ac, and protoxin Cry2Aa were provided by Dr. Marianne P. Carey (Case Western Reserve University, USA). Cry2Ab protoxin was provided by the Institute of Plant Protection, Chinese Academy of Agricultural Sciences (CAAS), China. Cry1A and Cry2A toxins were produced according to Monnerat *et al*. (1999).

Toxicological responses of each strain to Bt toxins were determined with diet overlay bioassays. Gradient concentrations of Bt toxin solution were prepared by diluting the toxin stock suspensions with a 0.01 mol/L, pH 7.4, phosphate‐buffered solution (PBS). Liquid artificial diet (900 *μ*L) was dispensed into each well (surface area = 2 cm^2^) of a 24‐well plate. After the diet cooled and solidified, 100 *μ*L of Bt toxin solution was applied evenly to the diet surface in each well. A single unfed neonate (24 h old) was put in each well after the toxin solution was dried at room temperature. Forty‐eight larvae were tested at each concentration of Bt toxins and PBS control. Mortality was recorded after 7 days, and larvae were considered as dead if they had died or weighed less than 5 mg.

The LC_50_ values (the concentration of Bt toxin killing 50% of larvae) and the 95% fiducial limits of the LC_50_ for each strain were calculated using the PoloPlus program (LeOra Software, 2002). Two LC_50_ values were considered significantly different if their 95% fiducial limits did not overlap.

### Design and preparation of single‐guide RNAs (sgRNAs)

The CRISPR target sites were screened on the open reading frame of *HaAPN1* (GenBank accession no. AY038607.1), *HaAPN2* (GenBank accession no. EU325551.1) and *HaAPN5* (GenBank accession no. AY038608.1) according to the target sequence principle: 5′‐GGN_18_NGG‐3′. *HaAPN1*, *HaAPN2* and *HaAPN5* were named according to the phylogenetic tree analysis based on lepidopteran APN protein sequences (Crava *et al*., 2010). Potential off‐target effects were checked by searching *H. armigera* genome (Pearce *et al*., 2017) through the basic local alignment search tool (BLAST). The target sequences were avoided if their final 12 nt (seed sequence) and NGG protospacer adjacent motif sequence matched with other genes (Cong *et al*., 2013).

The sgRNA was produced according to the method reported by Bassett *et al*. (2014). The specific forward oligonucleotides (sgRNA‐APN1‐F, sgRNA‐APN2‐F and sgRNA‐APN5‐F, the sequences of which are detailed in Table 1) were individually used with the universal reverse oligonucleotide (sgRNA‐R, its sequence is also detailed in Table 1) in a polymerase chain reaction (PCR) to synthesize template DNA. Reactions were performed with no template on an ABI thermal cycler at 98 °C 30 s, 35 cycles of (98 °C 10 s, 60 °C 30 s, 72 °C 15 s), 72 °C 10 min and 12 °C ∞. PCR products were purified by QIAquick^®^ PCR Purification Kit (QIAGEN, Hilden, Germany). *In vitro* transcription of each sgRNA was performed with MEGAshortscript^TM^ T7 High Yield Transcription Kit (Ambion, Foster City, CA, USA) according to the optimized manufacturer's instructions.

**Table 1:** Oligonucleotide sequences for *in vitro* preparation of the single‐guide RNA (sgRNA) templates and polymerase chain reaction primers for genotyping indel mutations of *HaAPN*s

Name	Sequence (5′–3′)†
sgRNA‐APN1‐F	GAAATTAATACGACTCACTATAGGTTCATGGAAACTTCGCCTGTTTTAGAGCTAGAAATAGC
sgRNA‐APN2‐F	GAAATTAATACGACTCACTATAGGTCTGGAGTAGACCCTGAAGTTTTAGAGCTAGAAATAGC
sgRNA‐APN5‐F	GAAATTAATACGACTCACTATAGGACAATACGCTCTAGAAGTGTTTTAGAGCTAGAAATAGC
sgRNA‐R	AAAAGCACCGACTCGGTGCCACTTTTTCAAGTTGATAACGGACTAGCCTTATTTTAACTTGCTATTTCTAGCTCTAAAAC
HaAPN1‐F	TTCCACACCACTCCCGAAACAT
HaAPN1‐R	GTCAAAAGTCCCCAGTTCTCCA
HaAPN2‐F	CAAGATACCCTCATCATGTCCA
HaAPN2‐R	GCCACCTTATCCATCTTCGG
HaAPN5‐F	TCAGCCTACTTGGTAACCTTCC
HaAPN5‐R	CTGAAGCCCAATAAGGAGAAGC

^†^The target sequences for each sgRNA are underlined.

### Egg collection and microinjection

Fresh eggs were washed down from the gauze in 1% sodium hypochlorite and rinsed with distilled water. After suction filtration, the eggs were lined up under a stereoscope and fixed on a coverslip with double‐sided adhesive tape for microinjection. About 1 nL of mixed sgRNA (500 ng/*μ*L) and Cas9 messenger RNA (mRNA) (500 ng/*μ*L) was injected into lined eggs according to the method reported by Wang *et al*. (2016). Cas9 mRNA (GeneArt™ CRISPR Nuclease mRNA) was purchased from Thermo Fisher Scientific (Shanghai, China). The injected eggs were maintained at 26 ± 1 °C, 60% ± 10% RH for hatching.

### Nondestructive genotyping of CRISPR/Cas9 induced indel mutations

Exuviates of the final instar larvae were used to prepare genomic DNAs to avoid damaging the pupae selected for genotyping as previously reported by Wang *et al*. (2016). DNA samples were prepared individually using AxyPrep Multisource Genomic DNA Miniprep Kit (Axygen, Hangzhou, China) according to the manufacturer's instructions. The fragment flanking the Cas9 target site for each *HaAPN* was amplified using a pair of specific primers (HaAPN1‐F/R, HaAPN2‐F/R, HaAPN5‐F/R, the sequences of which are detailed in Table 1). PCR products were directly sequenced. The PCR products showing multiple peaks in the sequence chromatogram were ligated into a pGEM‐T easy vector (Promega, Madison, WI, USA) and then sent to Life Technology (Shanghai, China) for monoclonal sequencing.

### Binding of Bt toxins with midgut brush border membrane vesicles (BBMVs)

Preparation of midgut BBMVs and binding of Bt toxins (Cry1Ac and Cry2Ab) with BBMVs were carried out as previously reported by Wang *et al*. (2017). Briefly, midguts of 1‐day‐old 5th‐instar larvae were dissected and 10 midguts were pooled for each strain. BBMVs were prepared and resuspended at a protein concentration of 1 mg/mL in a resuspension buffer (300 mmol/L Mannitol, 1 mmol/L dithiothreitol, 10 mmol/L Hepes‐Tris, pH 7.4). Prepared BBMV samples were stored at −80 °C until binding assays were conducted.

Twenty micrograms BBMVs with 0.04 nmol/L Cry1Ac toxin, or 5 *μ*g BBMVs with 0.28 nmol/L Cry2Ab toxin were incubated in 100 *μ*L binding buffer (8 mmol/L Na_2_HPO_4_, 2 mmol/L KH_2_PO_4_, 150 mmol/L NaCl, and 0.1% bovine serum albumin [BSA], pH 7.5) for 1 h at 37 °C. After incubation, BBMVs were pelleted by centrifugation at 16 000 × *g* and 4 °C for 10 min, and washed three times with 0.5 mL ice‐cold binding buffer to remove unbound toxins. The final pellet was resuspended in sodium dodecyl sulfate – polyacrylamide gel electrophoresis (SDS‐PAGE) sample buffer and boiled for 10 min. Denatured proteins were resolved on a 10% SDS‐PAGE gel and transferred to polyvinylidene difluoride (PVDF) filter. Filters were blocked with 5% BSA in PBST (pH 7.4, 25 mmol/L Tris, 3 mmol/L KCl, 135 mmol/L NaCl, 0.1% Tween 20) for 1 h. Toxin binding was revealed by Western blot using rabbit polyclonal antibodies of Cry1Ac or Cry2Ab (1 : 2000) (ABGENT, Suzhou, China) followed by horseradish peroxidase (HRP)‐conjugated goat anti‐rabbit immunoglobulin G (Sigma‐Aldrich, St. Louis, MO, USA) (1 : 5000) in PBST at room temperature for 1 h. Cry1Ac or Cry2Ab in the filter was visualized using SuperSignal^®^ West Pico Chemiluminescent Substrate (Rockford, IL, USA) according to the manufacturer's instructions.

## Results

### Construction of three homozygous APN‐knockout strains

**Figure 1:**
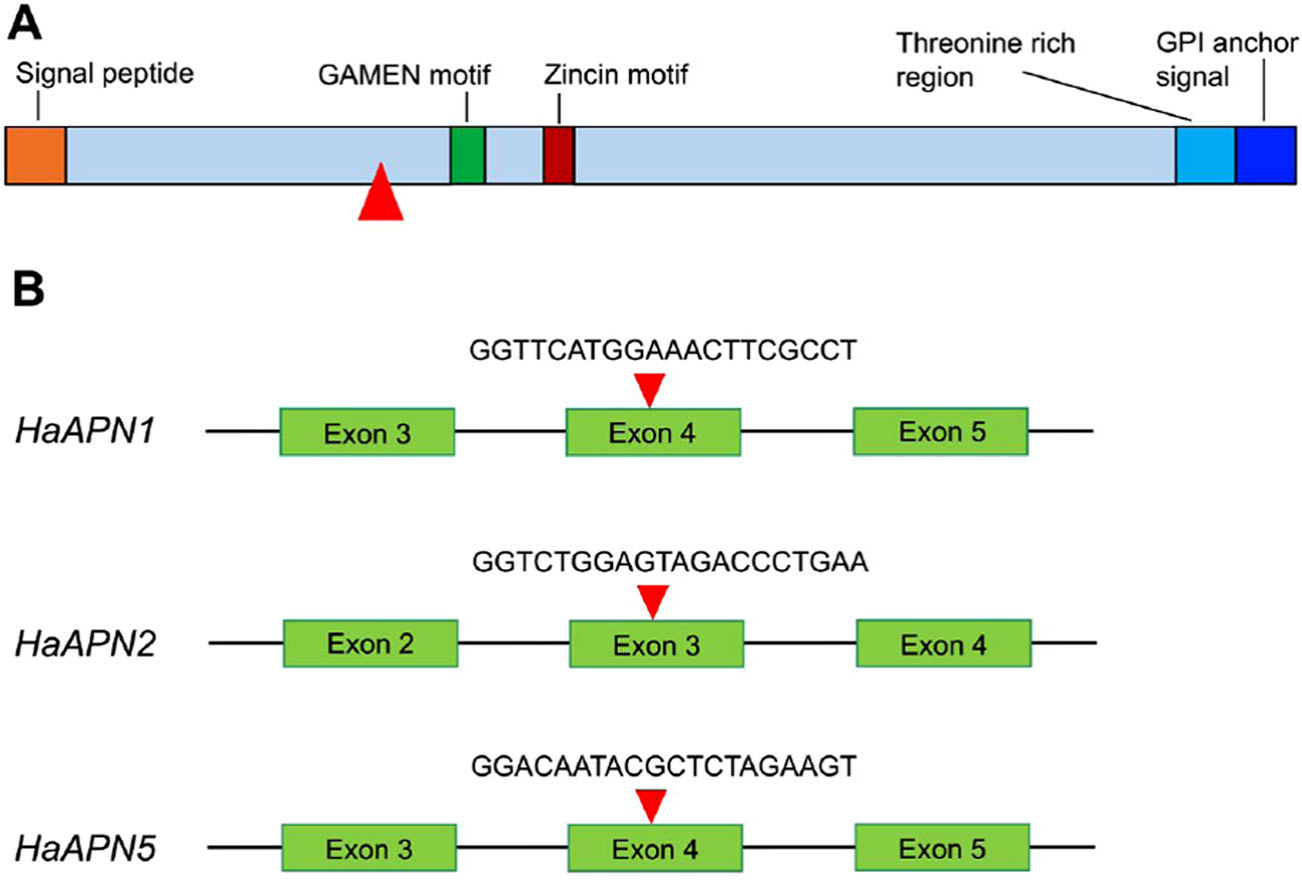
Schematic representation of an HaAPN (aminopeptidase N) protein of *Helicoverpa armigera* (A) and the positions and sequences targeted by single‐guide RNAs in *HaAPN1*, *HaAPN2* and *HaAPN5* (B). The cleavage sites of clustered regularly interspaced palindromic repeats (CRISPR)/CRISPR‐associated protein 9 are indicated by red triangles.

The sgRNAs were designed to target exon 4 of *HaAPN1* and *HaAPN5*, and exon 3 of *HaAPN2* respectively (Fig. 1, Table 1). If a frame shift mutation was created, it is expected the target APN will lose the critical functional domains including GAMEN motif, Zincin motif and GPI anchor signal region (Fig. 1A).

**Figure 2:**
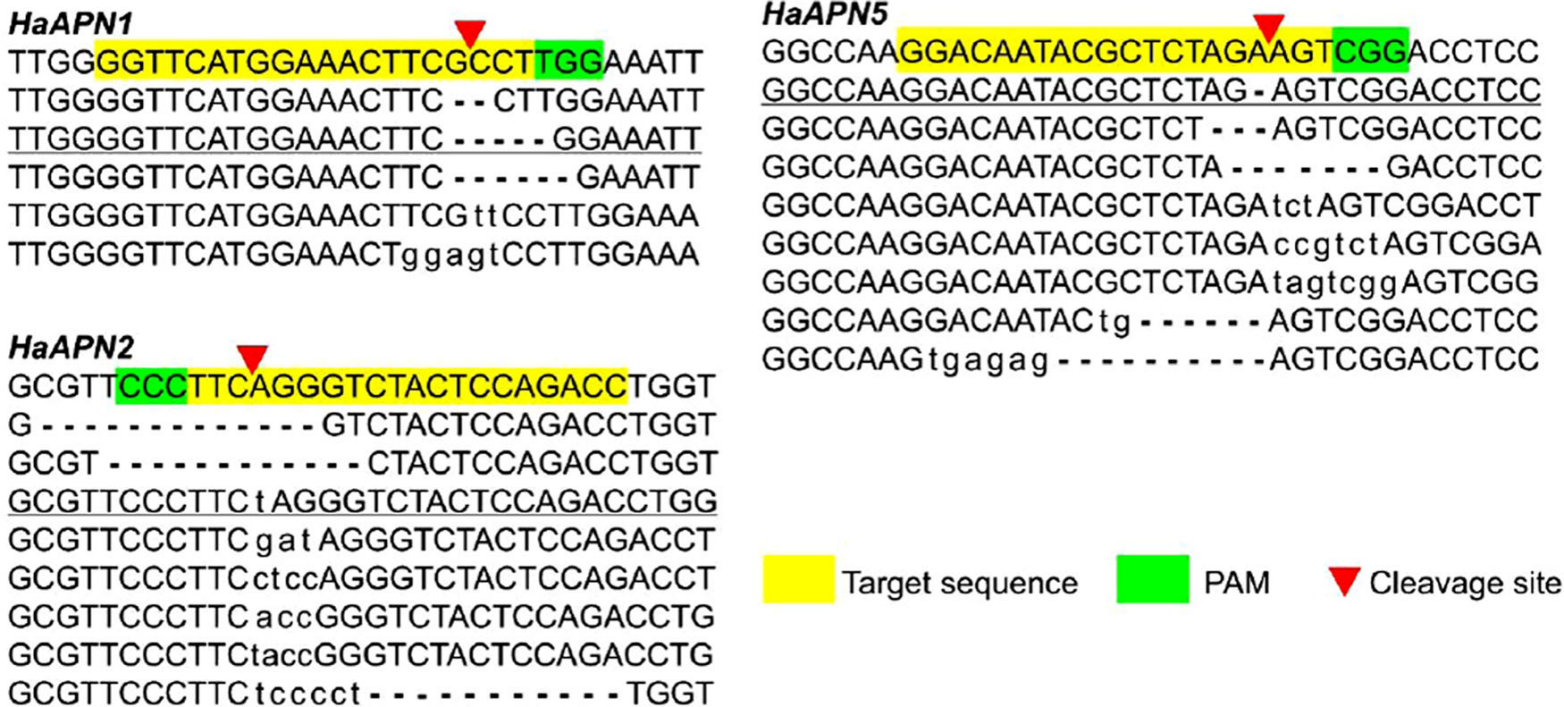
The clustered regularly interspaced palindromic repeats (CRISPR)/CRISPR‐associated protein 9 induced mutation types of the target *HaAPN* genes in G_0_. The target sequences of the wild type allele of each gene are highlighted in yellow and the protospacer adjacent motif (PAM) sequences in green. The cleavage site is shown as a red triangle. Deleted bases are represented by dashes and inserted bases are shown in lower case. The underlined mutation types for each *HaAPN* were subsequently selected to establish homozygous knockout strains.

About 1 nL of mixed sgRNA (500 ng/*μ*L) and Cas9 mRNA (500 ng/*μ*L) was injected into individual eggs from the SCD strain. The hatched larvae were reared to adults (G_0_) and mass crossed to produce the next generation (G_1_). Data of the number of eggs injected, hatching rate and *HaAPN* genotyping are detailed in Table 2. Genomic DNA of the G_0_ adults was prepared after oviposition, and sequencing results showed that 62.0% to 96.6% of the G_0_ individuals were edited for *HaAPN1*, *HaAPN2* and *HaAPN5*. The indel mutation types for each *HaAPN* gene are summarized in Figure 2. Larvae of G_1_ were reared to pupae and genotyped using the exuviates of the final instar larvae. To create the homozygous *HaAPN1* knockout strain, four heterozygotes (2♀ and 2♂) with a 5‐bp deletion allele (the major mutation type) and a wild type allele of *HaAPN1* were mass crossed to produce G_2_. In a similar way, 10 heterozygotes (5♀ and 5♂) with 1‐bp insertion in *HaAPN2* were mass crossed, and 14 heterozygotes (5♀ and 9♂) with 1‐bp deletion in *HaAPN5* were mass crossed to produce G_2_. Seventy to 96 individuals of G_2_ from the three populations were genotyped using exuviates of the final instar larvae, and only the individuals with homozygous indel mutations were pooled to generate homozygous knockout strains, named as SCD‐APN1, SCD‐APN2 and SCD‐APN5, respectively.

**Table 2:** The summary of genotyping data during the generation of *HaAPN* knockout strains

			Genotype (G_1_)§			
Target gene	Hatching rate (G_0_)†	Editing rate (G_0_)‡	MM	MW	WW	Homozygote frequency (G_2_)¶
*HaAPN1*	57.2% (95/166)	62.0% (31/50)	0	17	18	20.8% (20/96)
*HaAPN2*	37.1% (78/210)	96.6% (56/58)	53	44	39	11.5% (11/96)
*HaAPN5*	40.5% (81/200)	88.9% (48/54)	68	29	34	12.9% (9/70)

^†^Hatching rate = neonates / eggs injected.

^‡^Editing rate = moths edited / moths detected.

^§^MM: both *APN* alleles were mutated. MW: one *APN* allele was mutated and the other is the wild type. WW: both *APN* alleles were the wild type.

^¶^Homozygote frequency = homozygous mutants / pupae genotyped. *HaAPN1* mutation: 5‐bp deletion in exon 4; *HaAPN2* mutation: 1‐bp insertion in exon 3; *HaAPN5*: 1‐bp deletion in exon 4.

### Binding of Bt toxins to BBMVs from the APN knockouts and the SCD strain

**Figure 3:**
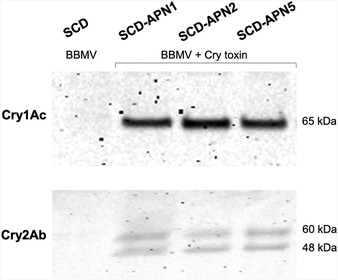
Qualitative binding of Cry1Ac and Cry2Ab to midgut brush border membrane vesicles (BBMVs). BBMVs from each strain were incubated with Cry1Ac or Cry2Ab, then pelleted by centrifugation. Bound toxins to pelleted BBMVs were separated by sodium dodecyl sulfate – polyacrylamide gel electrophoresis, blotted onto polyvinylidene difluoride membrane, and detected by either Cry1Ac or Cry2Ab antibodies. A single band of 65 kDa was observed for activated Cry1Ac, and two bands (60 kDa and 48 kDa) for activated Cry2Ab.

Qualitative *in vitro* binding of Cry1Ac and Cry2Ab to the midgut BBMVs was compared between the knockouts and the background SCD strain. Binding results showed that BBMVs from the three APN knockouts and the SCD strain can bind Cry1Ac and Cry2Ab (Fig. 3), suggesting individual knockout of *HaAPN1*, *HaAPN2* or *HaAPN5* did not result in substantial effects on binding capacity to Cry1Ac and Cry2Ab.

### Toxicological responses to Bt toxins of the APN knockouts and the SCD strain

Toxicological responses to Cry1A and Cry2A toxins were compared between the three HaAPN knockout strains and the background SCD strain. Bioassay results showed that none of the three knockouts had significant changes in susceptibility to Cry1A or Cry2A toxins when compared with the SCD strain, given the overlapped 95% fiducial limits between strains (see Table 3). Our results refuted the hypothesis that HaAPN1, HaAPN2 or HaAPN5 are mediators of Bt susceptibility in *H. armigera*, and suggested the three *HaAPN* genes we tested may not be important in the mode of action of Bt Cry1A or Cry2A toxins in *H. armigera*.

**Table 3:** Toxicological responses to Cry1A and Cry2A toxins in the wild‐type SCD strain and three knockouts of *Helicoverpa armigera*

Toxin	Strain	*N* †	Slope ± SE	LC_50_ (95% FL‡), *μ*g/cm^2^	TR§
Cry1Aa	SCD	384	1.7 ± 0.18	0.19 (0.15–0.24)	
	SCD‐APN1	384	1.7 ± 0.16	0.17 (0.13–0.21)	0.9
	SCD‐APN2	384	1.7 ± 0.16	0.16 (0.13–0.20)	0.8
	SCD‐APN5	384	1.7 ± 0.16	0.23 (0.19–0.29)	1.2
Cry1Ab	SCD	384	2.1 ± 0.28	0.043 (0.019–0.073)	
	SCD‐APN1	384	1.9 ± 0.18	0.035 (0.028–0.043)	0.8
	SCD‐APN2	384	1.7 ± 0.16	0.028 (0.019–0.039)	0.7
	SCD‐APN5	384	2.2 ± 0.20	0.041 (0.034–0.050)	1.0
Cry1Ac	SCD	384	1.9 ± 0.26	0.0038 (0.0030–0.0050)	
	SCD‐APN1	384	1.9 ± 0.18	0.0037 (0.0030–0.0045)	1.0
	SCD‐APN2	384	2.0 ± 0.19	0.0038 (0.0031–0.0046)	1.0
	SCD‐APN5	384	1.9 ± 0.18	0.0034 (0.0028–0.0042)	0.9
Cry2Aa	SCD	384	2.8 ± 0.35	0.42 (0.35–0.50)	
	SCD‐APN1	384	1.6 ± 0.15	0.27 (0.19–0.40)	0.6
	SCD‐APN2	384	1.7 ± 0.16	0.29 (0.23–0.36)	0.7
	SCD‐APN5	384	1.4 ± 0.14	0.30 (0.23–0.39)	0.7
Cry2Ab	SCD	384	2.0 ± 0.24	0.49 (0.39–0.62)	
	SCD‐APN1	384	1.7 ± 0.16	0.32 (0.26–0.40)	0.7
	SCD‐APN2	384	2.0 ± 0.18	0.38 (0.31–0.46)	0.8
	SCD‐APN5	384	1.6 ± 0.15	0.32 (0.25–0.40)	0.7

^†^Number of insects tested.

^‡^95% fiducial limits.

^§^Toxicity ratio = LC_50_ of the knockouts divided by LC_50_ of SCD.

LC_50_, concentration of toxin killing 50% of larvae.

## Discussion

The APN family consists of a class of enzymes that cleaves neutral amino acids from the N terminus of polypeptides. In the midgut of lepidopteran larvae, APNs work together with other peptidases to digest proteins ingested from host plants (Wang *et al*., 2005). Since an APN from *Manduca sexta* (MsAPN1) was first shown to bind to Cry toxins (Knight *et al*., 1995), many other *APNs* from different lepidopteran species have been cloned and exogenously expressed to show their ability to bind Cry1Ac toxin. However, few lepidopteran APNs have been identified as functional receptors for Cry toxins (Pigott & Ellar, 2007). *MsAPN1* from *M. sexta* was expressed in *Drosophila melanogaster*, and the transgenic flies showed increased susceptibility to Cry1Ac (Gill & Ellar, 2002), which identified MsAPN1 as a functional receptor for Cry1Ac. Later, a RNAi‐based gene silencing study confirmed the important role of MsAPN1 in the mode of action of Cry1Ac (Floes‐Escobar *et al*., 2013). These two studies have provided the most compelling evidence so far to verify an APN as a functional receptor for Bt toxin.

RNAi‐mediated gene silence strategy has been used to understand biological relevance of APNs to the mode of action of Bt toxins, and several lepidopteran APNs have been suggested as putative receptors for Cry1A, Cry1C or Cry2A toxins (Rajagopal *et al*., 2002; Sivakumar *et al*., 2007; Flores‐Escobar *et al*., 2013; Wang *et al*., 2017; Zhao *et al*., 2017; Qiu *et al*., 2017a, 2017b). Because of the low interference efficiency of RNAi in lepidopteran insects (Terenius *et al*., 2011), we need to be cautious when the RNAi technique is utilized for functional validation of candidate APNs as Bt receptors in these species. Therefore, further evidence is needed to clarify the functional role of these APNs as Bt receptors.

CRISPR/Cas9 genome editing is a useful technology for functional validation of candidate receptor genes *in vivo* for Bt toxins. This tool has been successfully adopted to confirm the *HaCad* and *HaABCA2* of *H. armigera* as functional receptors for Cry1Ac and Cry2Ab, respectively (Wang *et al*., 2016, 2017). In the present study, we found binding of Cry1Ac and Cry2Ab to midgut BBMVs was not obviously affected by knockout of *HaAPN1*, *HaAPN2* or *HaAPN5*. Meanwhile, no significant difference in susceptibility to Cry1A and Cry2A toxins was observed between the three knockout strains and the control strain. Our results show that HaAPN1, HaAPN2 or HaAPN5 of *H. armigera* are not important in mediating susceptibility to Cry1A or Cry2A toxins tested. Because there are 10 APNs in the genome of *H. armigera* (Angelucci *et al*., 2008; Pearce *et al*., 2017), our results do not exclude the possibility of one or more of the other seven APNs functioning as Bt receptors.

Down‐regulation of *APN1* has been associated with resistance to Cry1A toxins in other lepidopteran species. Reduced expression of an *APN* of *Trichoplusia ni* (*TnAPN1*) was genetically linked with Cry1Ac resistance, suggesting TnAPN1 as a functional receptor of Cry1Ac (Tiewsiri & Wang, 2011). Down‐regulation of *APN1* of *Ostrinia nubilalis* was genetically associated with Cry1Ab resistance, also suggesting OnAPN1 is a critical receptor in the Cry1Ab mode of action (Coates *et al*., 2013). It will be interesting to knock out these two APN genes with the CRISPR/Cas9 approach to see if the knockouts confer resistance to their corresponding Bt toxins.
